# Morphological and molecular description of *Rhadinorhynchus laterospinosus* Amin, Heckmann & Ha, 2011 (Acanthocephala, Rhadinorhynchidae) from marine fish off the Pacific coast of Vietnam

**DOI:** 10.1051/parasite/2019015

**Published:** 2019-03-06

**Authors:** Omar Mohamed Amin, Richard Anderson Heckmann, Sara Dallarés, María Constenla, Nguyen Van Ha

**Affiliations:** 1 Institute of Parasitic Diseases 11445 E. Via Linda 2-419 Scottsdale AZ 85259 USA; 2 Department of Biology, Brigham Young University 1114 MLBM Provo UT 84602 USA; 3 Department of Animal Biology, Vegetal Biology and Ecology, Universitat Autònoma de Barcelona Cerdanyola 08193 Barcelona Spain; 4 Department of Parasitology, Institute of Ecology and Biological Resources (IEBR), Vietnam Academy of Science and Technology 18 Hoang Quoc Viet Cau Giay Hanoi Vietnam

**Keywords:** Acanthocephala, *Rhadinorhynchus laterospinosus*, Host distribution, Molecular profile, EDAX, Micropores, Vietnam

## Abstract

*Rhadinorhynchus laterospinosus* Amin, Heckmann & Ha, 2011 (Rhadinorhynchidae) was described from a single female collected from a trigger fish, *Balistes* sp. (Balistidae) from the northern Pacific coast of Vietnam in Halong Bay, Gulf of Tonkin. More recent collections of fishes in 2016 and 2017 revealed wider host and geographical distributions. We report this Acanthocephala from nine species of fish representing six families (including the original record from *Balistes* sp.) along the whole Pacific coast of Vietnam. The fish species are *Alectis ciliaris* (Carangidae), *Auxis rochei* (Scombridae), *Auxis thazard* (Scombridae), *Leiognathus equulus* (Leiognathidae), *Lutjanus bitaeniatus* (Lutjanidae), *Megalaspis cordyla* (Carangidae), *Nuchequula flavaxilla* (Leiognathidae), and *Tylosurus* sp. (Belonidae). We provide a complete description of males and females of *R. laterospinosus*, discuss its hook metal microanalysis using EDAX, and its micropores. Specimens of this species characteristically have lateral trunk spines bridging the anterior ring of spines with posterior field of ventral spines and a proboscis with 15–19 longitudinal alternating rows of 21–26 hooks each varying with host species. We demonstrate the effect of host species on the distribution and size of the trunk, proboscis, proboscis hooks, trunk spines, and reproductive structures. The molecular profile of this acanthocephalan, based on 18S rDNA and *cox*1 genes, groups with other *Rhadinorhynchus* species and further seems to confirm the paraphyly of the genus, which is discussed.

## Abstract (fr)

*Rhadinorhynchus laterospinosus* Amin, Heckmann & Ha, 2011 (Rhadinorhynchidae) a été décrit d’une seule femelle prélevée chez *Balistes* sp. (Balistidae) sur la côte nord du Pacifique du Vietnam, dans la Baie d’Along, golfe du Tonkin. Des collections de poissons plus récentes, en 2016 et 2017, ont révélé une répartition en hôtes et en localités géographiques plus étendue. Nous rapportons cet Acanthocéphale de 9 espèces de poissons représentant 6 familles (y compris la mention originale chez *Balistes* sp.), le long de la côte pacifique du Vietnam. Les espèces de poissons sont *Alectis ciliaris* (Carangidae), *Auxis rochei* (Scombridae), *Auxis thazard* (Scombridae), *Leiognathus equulus* (Leiognathidae), *Lutjanus bitaeniatus* (Lutjanidae), *Megalaspis cordyla* (Carangidae), *Nuchequula flavaxilla* (Leiognathidae) et *Tylosurus* sp. (Belonidae). Nous fournissons une description complète des mâles et des femelles de *R. laterospinosus*, de la microanalyse des métaux des crochets à l’aide d’EDAX, et de ses micropores. Les spécimens de cette espèce ont généralement des épines latérales du tronc reliant l’anneau antérieur d’épines avec un champ postérieur d’épines ventrales et un proboscis avec 15–19 rangées longitudinales alternées de 21 à 26 crochets, variant avec l’espèce hôte. Nous démontrons l’effet des espèces hôtes sur la distribution et la taille du tronc, du proboscis, des crochets du proboscis, des épines du tronc et des structures de reproduction. Le profil moléculaire de cet acanthocéphale, basé sur les gènes de l’ADNr 18S et de *cox1*, forme un groupe avec d’autres espèces de *Rhadinorhynchus* et semble en outre confirmer la paraphylie du genre, ce qui est discuté.

## Introduction

Most of the recent taxonomic work on the Acanthocephala from Vietnam has been reported by the Amin-Heckmann-Ha team since 2000. A number of acanthocephalan species from freshwater and marine fish, amphibians, reptiles, birds, and mammals were previously described in Vietnam [3, 9–13, 16]. Additionally, 11 species of acanthocephalans were collected from marine fish off the eastern seaboard of Vietnam in Halong Bay in 2008 and 2009. Of these, six new species of *Neoechinorhynchus* Stiles & Hassall 1905, one new species of *Heterosentis* Van Cleave, 1931, and two new species of *Rhadinorhynchus* Lühe 1911 were described [8, 14, 15]. Four other species of Echinorhynchid acanthocephalans from marine fishes in Halong Bay were described [4] and five other new species from fishes and amphibians of eight collected host species were also described. Three other species of *Rhadinorhynchus* and one species of *Gorgorhynchus* were otherwise previously reported from marine fishes in Vietnam by other observers [19].

Fifteen species of acanthocephalans in five families were more recently collected from fishes on the Pacific coast and amphibians in central Vietnam in 2016 and 2017. In the present report, we describe males and females of *R. laterospinosus*, which was originally described from a single female specimen, from extensive collections of fishes along the Pacific coast of Vietnam and provide a molecular profile of that species based on small subunit ribosomal DNA (18S rDNA) and partial mitochondrial cytochrome c oxidase 1 (*cox*1) genes. Furthermore, its phylogenetic relationships with other *Rhadinorhynchus* and closest-related species are analyzed and discussed.

## Materials and methods

### Collections

Collections of 215 specimens of *R. laterospinosus* from nine species of fish in six families in 2016 and 2017 along the Pacific coast of Vietnam are detailed in Table 1 along with infection parameters, geographical locations and museum numbers of deposited material at the Harold W. Manter Laboratory, Nebraska State Museum, Lincoln, Nebraska.

**Table 1:** Host and geographic distribution of *Rhadinorhynchus laterospinosus* in the Pacific Ocean off Vietnam.

Hosts	No. exam.	No. infect. (%)	Specimens (mean)	Date of collection	Location (North, South)	Coordinates	HWML coll. no.
*Alectis ciliaris* (Bloch) (Carangidae) African pompano	10	1 (10)	1 (0.1)	May, 2016	Nha Trang (S)	12°15′N, 109°11′E	139,496
*Auxis rochei* (Lacépède) (Scombridae) Bullet tuna*	10	7 (70)	101 (10.1)	May, 2016	Nha Trang (S)	12°15′N, 109°11′E	139,488–139,491
*Auxis thazard* (Lacépède) (Scombridae) Frigate tuna*	14	11 (79)	114 (8.1)	Jan, 2016, Oct, 2017	Nha Trang (S)	12°15′N, 109°11′E	139,494, 139,495
*Balistes* sp. (Linn.); type host (Balistidae) Trigger fish	2	1 (50)	1 (0.5) holo.	May, 2009	Halong Bay (N)	20°51′54.5″N, 106°41′01.8″E	49,298
*Leiognathus equulus* Forsskal (Leiognathidae) Common pony fish	11	3 (27)	10 (0.9) allo.	Jan, 2016	Hai Phong (N)	20°51′54.5″N, 106°41′01.8″E	139,486
					Nha Trang (S)	12°15′N, 109°11′E	139,487
*Lutjanus bitaeniatus* (Valenciennes) (Lutjanidae) Indonesian snapper	3	1 (33)	1 (0.3)	May, 2016	Nha Trang (S)	12°15′N, 109°11′E	139,492
*Megalaspis cordyla* (Linn.) (Carangidae) Torpedo scad	2	1 (50)	1 (0.5)	May, 2017	Quang Binh (N)	17°30′N, 106°20′E	139,497
*Nuchequula flavaxilla* Kimura, Kimura, Ikejuma (Leiognathidae) Yellow-spotted pony fish	20	7 (35)	10 (0.5)	March, 2017	Quang Ninh (N)	21°15′N, 107°20′E	139,493
*Tylosurus* sp. (Cocco) (Belonidae) Needle fish	2	2(100)	2 (1.0)	?	Binh Thuan (S)	10°56′N, 108°6′E	
Total	74	34 (46)	215 (2.9)				

* Measurements were mostly based on specimens from these two hosts.

### Methods

Freshly collected acanthocephalans were extended in water until proboscides were everted and fixed in 70% ethanol for transport to our Institute of Parasitic Diseases (IPD) in Arizona, USA for processing and further studies. Worms were punctured with a fine needle and subsequently stained in Mayer’s acid carmine, destained in 4% hydrochloric acid in 70% ethanol, dehydrated in ascending concentrations of ethanol reaching 100% (24 h each), and cleared in 100% xylene then in 50% Canada balsam and 50% xylene (24 h each). Whole worms were then mounted in Canada balsam. Measurements are in micrometers, unless otherwise noted; the range is followed by the mean values between parentheses. Width measurements represent maximum width. Trunk length does not include proboscis, neck, or bursa.

Line drawings were created by using a Ken-A-Vision micro-projector (Ward’s BiologicalSupply Co., Rochester, New York), which uses cool quartz iodine 150 W illumination with 10×, 20×, and 43× objective lenses. Images of stained whole mounted specimens were projected vertically on 300 series Bristol draft paper (Starthmore, Westfield, Massachusetts), then traced and inked with India ink. Projected images were identical to the actual specimens being projected.

Specimens were deposited in the University of Nebraska’s State Museum’s Harold W. Manter Laboratory (HWML) collection in Lincoln, Nebraska, USA. Accession numbers are noted in Table 1.

### Scanning electron microscopy (SEM)

About 15 specimens from four host species that had been fixed and stored in 70% ethanol were processed for SEM following standard methods [36]. These included critical point drying (CPD) in sample baskets and mounting on SEM sample mounts (stubs) using conductive double sided carbon tape. Samples were coated with gold and palladium for 3 min using a Polaron #3500 sputter coater (Quorum (Q150 TES) www.quorumtech.com) establishing an approximate thickness of 20 nm. Samples were placed and observed in an FEI Helios Dual Beam Nanolab 600 (FEI, Hillsboro, Oregon) Scanning Electron Microscope, with digital images obtained in the Nanolab software system (FEI, Hillsboro, Oregon) and then stored on a USB for future reference. Samples were received under low vacuum conditions using 10 kV, spot size 2, 0.7 Torr using a GSE detector.

### EDXA (energy dispersive X-ray analysis)

Standard methods were used for preparation, similar to the SEM procedure. Eight specimens were examined and positioned with the above SEM instrument which was equipped with a Phoenix energy-dispersive X-ray analyzer (FEI, Hillsboro, Oregon). X-ray spot analysis and live scan analysis were performed at 16 kV with a spot size of five and results were recorded on charts and stored with digital imaging software attached to a computer. The TEAM *(Texture and Elemental Analytical Microscopy) software system (FEI, Hillsboro, Oregon) was used. Data were stored on a USB. The data included weight percent and atom percent of the detected elements, following correction factors, and were stored on a USB. All figures on the USB can be viewed by contacting the second author. The hooks were cut and scanned at two positions (tip and middle) with a gallium beam (LIMS) using a dual beam scanning electron microscope. The alignment of the hook previous to cutting generated a cross section of the area.

### Ion sectioning of hooks

A dual-beam SEM with a gallium (Ga) ion source (GIS) was used for the LIMS (Liquid Ion Metal Source) part of the process. The gallium beam (LIMS) is a gas injection magnetron sputtering technique whereby the rate of cutting can be regulated. The hooks of six acanthocephalans were centered on the SEM stage and cross-sectioned using a probe current between 0.2 nA and 2.1 nA according to the rate at which the area is cut. The time of cutting is based on the nature and sensitivity of the tissue. Following the initial cut, the sample also goes through a milling process to obtain a smooth surface. The cut was then analyzed with X-ray at the tip, middle, and base of hooks for chemical ions with an electron beam (Tungsten) to obtain an X-ray spectrum. Results were stored with the attached imaging software then transferred to a USB for future use. The intensity of the GIS was variable according to the nature of the material being cut.

### Molecular methods

Total genomic DNA was extracted from four specimens of *R. laterospinosus* from *Auxis rochei* preserved in 70% ethanol using a Qiagen™ (Valencia, California, USA) DNeasy^®^ Tissue Kit, and following the manufacturer’s instructions. Partial nuclear small subunit ribosomal DNA (18S rDNA) and partial fragments of mitochondrial cytochrome c oxidase 1 (*cox1*) gene were amplified (50 μL total volume) using ExcelTaq^TM^ SMOBIO^®^ PCR Master Mix (Taiwan) containing: 5× concentrated master mix, that is, a mixture of recombinant Taq DNA polymerase, reaction buffer, MgCl_2_ (2 mM), dNTPs (0.2 mM), and enzyme stabilizer; 0.25 μM of each PCR primer and 2 μL of extracted gDNA. Primer pairs and amplification conditions used were as follows.

Partial fragments of the 18S rDNA gene were amplified using the primers 18SU467F (forward, 5′-ATCCAAGGAAGGCAGCAGGC-3′) and 18SL1310R (reverse, 5′-CTCCACCAACTAAGAACGGC-3′) [46] under the following thermocycling conditions: initial denaturation at 94 °C for 3 min followed by 40 cycles (denaturation for 30 s at 94 °C, annealing for 45 s at 56 °C, and extension for 2 min at 72 °C), and a final extension step at 72 °C for 7 min.

Partial fragments of the *cox*1 gene were amplified using the primers LCO1490 (forward, 5′-GGTCAACAAATCATAAAGATATTGG-3′) and HCO2198 (reverse, 5′-TAAACTTCAGGGTGACCAAAAAATCA-3′) [23] under the following thermocycling conditions: initial denaturation at 95 °C for 15 min followed by 40 cycles (denaturation for 5 min at 80 °C, followed by 1 min 30 s at 92 °C, annealing for 1 min at 42 °C, and extension for 2 min at 72 °C), and a final extension step at 72 °C for 10 min.

In every PCR run, a negative and a positive control were used to detect any potential contamination and to have a reliable sample to compare with, respectively. PCR amplicons were sequenced directly for both strands using the same PCR primers.

Sequences were assembled and edited using Mega v6 [47] and submitted to GenBank under accession numbers: MK457183 – MK457185 (18S) and MK572741–MK572744 (*cox*1). Sequences were aligned using Muscle as implemented in MEGA v6 together with published sequences of *Rhadinorhynchus* and most closely-related published sequences to members of this genus. *Rotaria rotatoria* (Pallas, 1776) was used as the outgroup in both the 18S (DQ089736) and *cox*1 (EU499879) datasets. Both alignments (18S: 760 nt positions of which eight were excluded prior to analysis; *cox*1: 537 nt positions of which 26 were excluded prior to analysis) were used for comparative sequence analysis.

The SeaView v4 interface [27] was used to select blocks of evolutionarily conserved sites. Maximum likelihood (ML) and Bayesian inference (BI) algorithms were used for phylogenetic tree reconstruction after determination of the best-fit model of nucleotide substitution with jModelTest v2.1.4 [22] using the Akaike Information Criterion (AIC) and the Bayesian Information Criterion (BIC), respectively. For the ML algorithm, the best-fitting model selected was the GTR + G model (nst = 6, rates = gamma, ngammacat = 4) both for the 18S and *cox*1 datasets. In the case of BI, the best-fitting model was TVMef + G (nst = mixed, rates = gamma, ngammacat = 4) for the 18S dataset and TrN + G (nst = 6, rates = gamma, ngammacat = 4) for the *cox*1 dataset. ML analyses were performed in PhyML v3.0 [30] with a non-parametric bootstrap of 100 replicates. BI analyses were carried out with MrBayes v3.2.6 [42] on the CIPRES Science Gateway v3.3 [39]. Log likelihoods were estimated over 10,000,000 generations using Markov Chain Monte Carlo (MCMC) searches on two simultaneous runs of four chains, sampling trees every 1000 generations. The first 25% of the sampled trees were discarded as “burn-in” and a consensus topology and nodal support estimated as posterior probability values [35] were calculated from the remaining trees. Pairwise genetic distance matrices were calculated using the “uncorrected *p*-distance” model implemented in MEGA v6.

## Results

*Rhadinorhynchus laterospinosus* was originally described from one female specimen collected from an individual triggerfish, *Balistes* sp. (Linn.) (Balistidae), from the Pacific coast at Halong Bay in May of 2009. It has since been found in eight other species of fish in five other families along the Pacific coast of Vietnam from the north at Hai Phong and Quang Binh to the south at Nha Trang and Binh Thuan (Table 1). We have studied specimens from all host species but provide measurements of specimens from the more extensive collections from two hosts, *Auxis rochei* (Lacépède) and *Auxis thazard* (Lacépède). The description is inclusive of morphometric differences noted between specimens collected from these two-host species (Table 2).

**Table 2:** The relationship between host species and size of certain anatomical structures of measured specimens of *Rhadinorhynchus laterospinosus* collected off the Pacific coast of Vietnam in 2016.

Worm sex	Character	Host species			
		*Auxis thazard* (*n* = 12 males, 15 females)		*Auxis rochei* (*n* = 18 males, 18 females)	
Male	Trunk length (mm)	5.75–11.25 (8.09)		4.75–8.37 (6.33)	
Female	Trunk length (mm)	7.80–26.25 (16.01)		8.00–21.25 (13.16)	
Male	No. ant. trunk spines in row (dorsal, mid, vent.)*	1–3 (2), 2–4 (3), 1–2 (2)		1–3 (2), 2–4 (3), 1–2 (2)	
Female	No. ant. trunk spines in row (dorsal, mid, vent.)	1–4 (2), 1–4 (3), 2–4 (3)		2–4 (3), 3–5 (4), 2–5 (3)	
Male	No. post. trunk spines in row (vent., lateral)	1–4 (3), 5–12 (8)		0–8 (3), 2–23 (9)	
Female	No. post. trunk spines in row (vent., lateral)	0–13 (6), 1–19 (11)		5–16 (10), 5–31 (17)	
Male	L of ant. trunk spines (dorsal, mid, vent.)	42–62 (50), 31–42 (36), 21–63 (42)		31–73 (51), 31–52 (43), 31–62 (46)	
Female	L of ant. trunk spines (dorsal, mid, vent.)	52–86 (64), 42–73 (54), 51–62 (56)		48–72 (60), 31–62 (48), 46–61 (53)	
Male	L of ventral post. trunk spines (ant., mid, post.)	40–62 (50), 42–66 (52), 20–42 (37)		32–73 (54), 51–83 (63), 40–72 (64)	
Female	L of ventral post. trunk spines (ant., mid, post.)	42–81 (63), 62–96 (79), 42–73 (59)		52–84 (64), 61–95 (78), 51–95 (67)	
Male	L of lateral post. trunk spines (ant., mid, post.)	22–32 (29), 31–52 (44), 31–43 (37)		41–52 (40), 40–72 (47), 30–62 (40)	
Female	L of lateral post. trunk spines (ant., mid, post.)	31–72 (57), 63–86 (65), 42–70 (54)		41–73 (52), 62–82 (68), 41–73 (55)	
Female	Proboscis length (mm)	1.25–1.82 (1.55)		1.45–1.90 (1.71)	
	Proboscis hook rows	15–17 (16.4)		15–19 (17.3)	
	Hook length	Dorsal	Ventral	Dorsal	Ventral
Male	Apical prob. hook L	37–40 (39) × 7–10 (9)	42–50 (46) × 11–12 (12)	35–47 (39) × 7–11 (10)	37–50 (44) × 11–12 (11)
Male	Subapical prob. hook L × W	45–55 (51) × 10–15 (12)	52–62 (58) × 12–17 (15)	42–60 (53) × 10–12 (12)	47–63 (55) × 12–15 (13)
Male	Mid prob. hook L × W	55–60 (58) × 12–14 (13)	60–67 (65) × 14–18 (16)	52–62 (59) × 12	60–77 (67) × 13–18 (15)
Male	Post. prob. hook L × W	35–37 (36) × 8–11 (10)	37–47 (42) × 11–13 (12)	27–42 (35) × 7–10 (8)	35–50 (41) × 9–12 (11)
Male	Basal prob. hook L × W	50–60 (53) × 11–15 (13)	56–72 (62) × 13–17 (15)	47–55 (51) × 10–14 (11)	55–72 (63) × 12–17 (14)
Female	Apical prob. hook L × W	50–60 (54) × 10–12 (11)	52–65 (59) × 11–15 (13)	45–52 (48) × 10–15 (12)	45–60 (53) × 12–15 (14)
Female	Subapical prob. hook L × W	60–65 (62) × 13–17 (15)	63–70 (66) × 15–17 (17)	62–72 (65) × 12–20 (15)	60–70 (66) × 14–20 (16)
Female	Mid prob. hook L × W	70–77 (73) × 13–17 (15)	73–80 (77) × 20–22 (21)	70–77 (73) × 12–15 (14)	75–78 (76) × 18–20 (19)
Female	Post. prob. hook L × W	40–45 (42) × 10–11 (10)	42–50 (48) × 10–15 (12)	32–45 (40) × 10–12 (11)	50–57 (54) × 10–15 (13)
Female	Basal prob. hook L × W	65–72 (68) × 10–15 (12)	72–82 (75) × 15–17 (16)	57–82 (68) × 12–17 (14)	75–87 (79) × 12–18 (16)
Male	Prob. Recept. L × W (mm)	2.08–3.45 (2.45) × 0.14–0.35 (0.25)		1.62–2.62 (2.28) × 0.15–0.30 (0.21)	
Male	Anterior testis (mm)	0.69–1.62 (1.19) × 0.31–0.50 (0.40)		0.59–1.75 (0.98) × 0.22–0.52 (0.35)	
Male	Posterior testis (mm)	0.52–1.25 (0.99) × 0.25–0.57 (0.42)		0.47–1.50 (0.84) × 0.22–0.47 (0.33)	
Male	Ant. cement glands (mm)	0.78–1.04 (0.95) × 0.17–0.32 (0.24)		0.31–1.09 (0.64) × 0.10–0.27 (0.18)	
Male	Post. cement glands (mm)	0.78–1.25 (1.05) × 0.17–0.26 (0.21)		0.36–1.09 (0.62) × 0.14–0.26 (0.16)	
Male	Saefftigen’s pouch (mm)	0.83–1.27 (1.08) × 0.18–0.27 (0.22)		0.42–1.00 (0.78) × 0.15–0.26 (0.20)	

* All observations of trunk spines are made on one side of the trunk.

### *Rhadinorhynchus laterospinosus* Amin, Heckmann, Ha, 2011

Family: Rhadinorhynchidae Travassos, 1923

Genus: *Rhadinorhynchus* Lühe 1911

Type host: Triggerfish *Balistes* sp. (Linn.) (Balistidae).

Other hosts: See Table 1.

Type locality: Halong Bay (20°51′N, 54.5″E).

Other localities along the Pacific coast of Vietnam: See Table 1.

Specimens: HWML collection no. 49,298 (holotype female) Amin et al., 2011; no. 139,486 (allotype male); nos. 139,487–139,497 of many paratypes from eight other host species in Table 1.

#### Description

**Figures 1–9:**
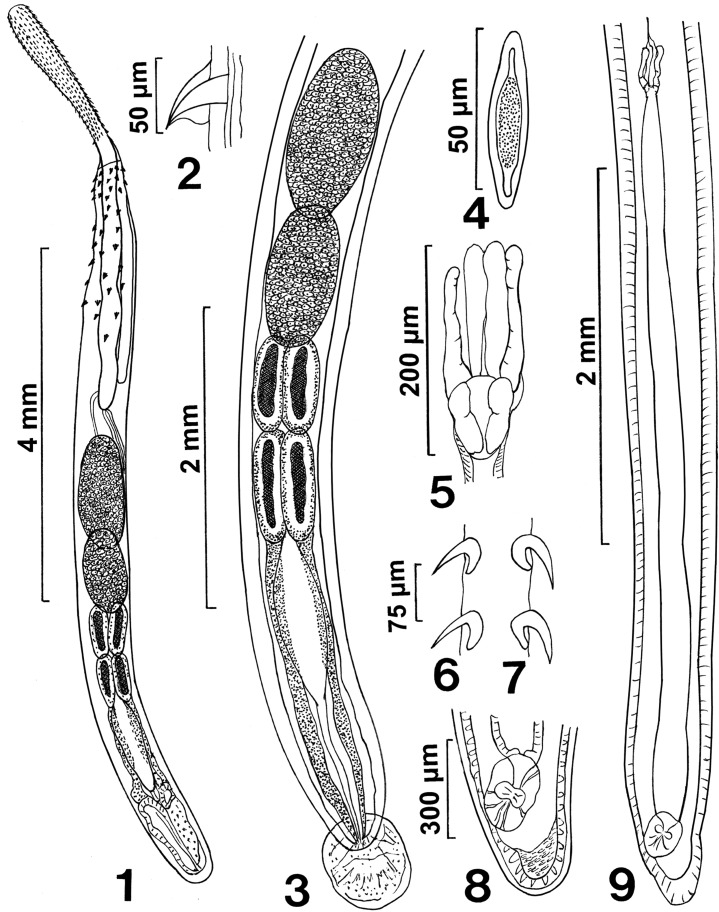
Line drawings of whole mounted specimens of *Rhadinorhynchus laterospinosus* from *Auxis rochei* and *Auxis thazard* in the Pacific Ocean off Vietnam. (1) A paratype male showing the anteriorly enlarged proboscis, the distribution of trunk spines within the range of the long receptacle, and the posterior distribution of the reproductive system. (2) A posterior ventral trunk spine of a female specimen. (3) Detailed male reproductive system. Note the large tubular giant nuclei of the cement glands and the posterior extension of the cement gland ducts surrounding Saefftigen’s pouch anteriorly. (4) A ripe egg. (5) Detail of the uterine bell of the female specimen shown in Figure 9. Note the inner paired rod-like structures. (6, 7) Dorsal (Fig. 6) and ventral (Fig. 7) hooks at the mid proboscis of a female specimen. Note differences in the thickness, length, and curvature of dorsal vs. ventral hooks. (8) Detail of the vagina from Figure 9. Note the inner muscular plug lining of the posterior tip of the trunk. (9) A complete female reproductive system characterized by the long and wide uterus.

**Figures 10–15:**
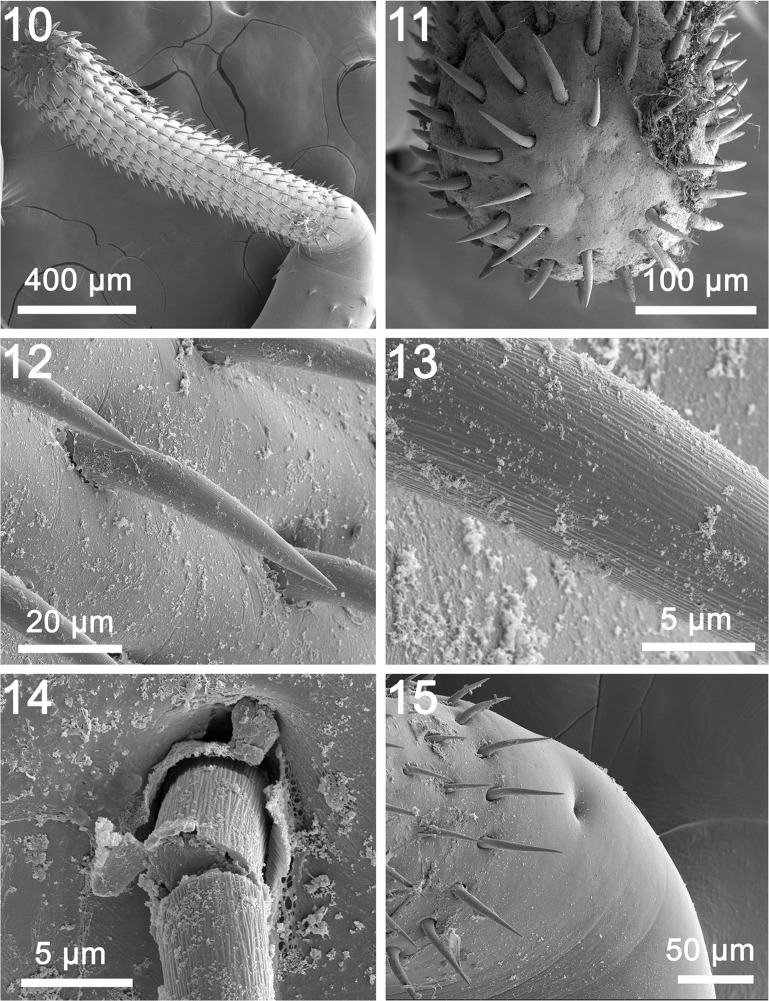
SEM of specimens of *Rhadinorhynchus laterospinosus* from *Auxis rochei* and *Auxis thazard* in the Pacific Ocean off Vietnam. (10) The proboscis of a female specimen. (11) The apical end of the proboscis in Figure 10 showing the smaller apical hooks, the organization of hook rows and no external evidence of an apical organ. (12) A typical example of hook shape and orientation from the midsection of a proboscis. (13) A magnified view of a hook showing its surface serrations. (14) A broken hook demonstrating its thick core and thin cortical layer. (15) Posterior end of a proboscis showing the larger hooks in the posterior circle and a sensory pit.

**Figures 16–21:**
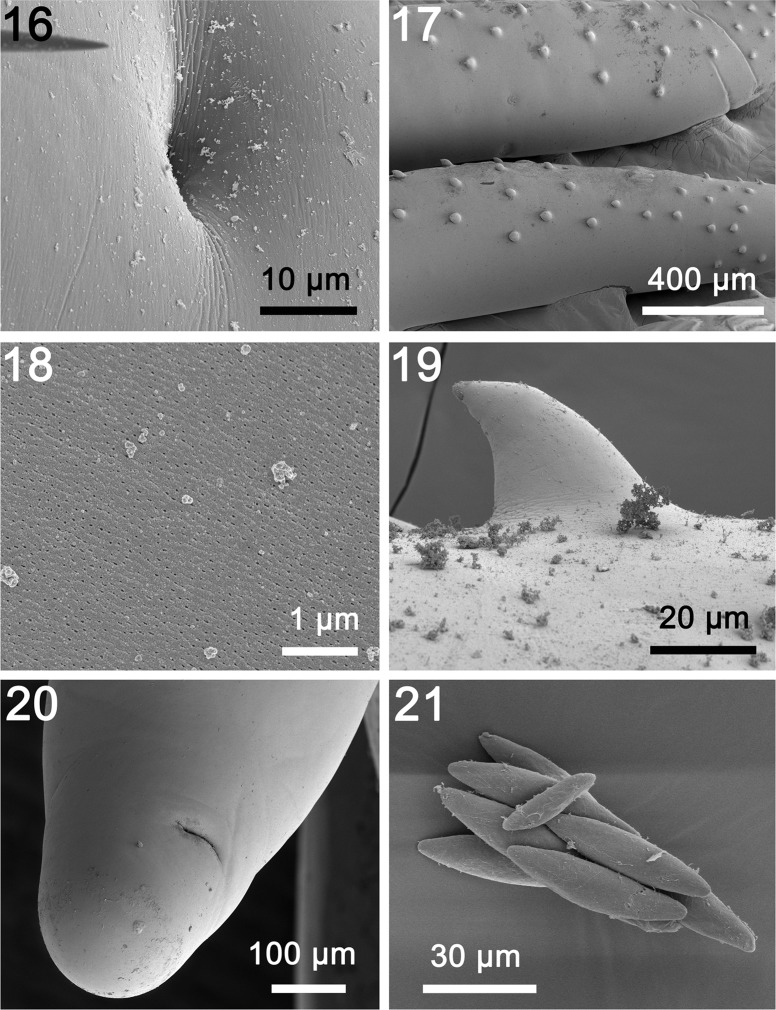
SEM of specimens of *Rhadinorhynchus laterospinosus* from *Auxis rochei* and *Auxis thazard* in the Pacific Ocean off Vietnam. (16) A larger magnification of the neck sensory pit showing no rim outline. (17) The anterior end of two specimens showing the posterior zone of ventral and lateral spines. (18) Micropores at the anterior part of the trunk. (19) A high magnification of a trunk spine. (20) The bluntly pointed posterior end of a female specimen showing the sub-ventral gonopore. (21) A small cluster of eggs.

**Figures 22–27:**
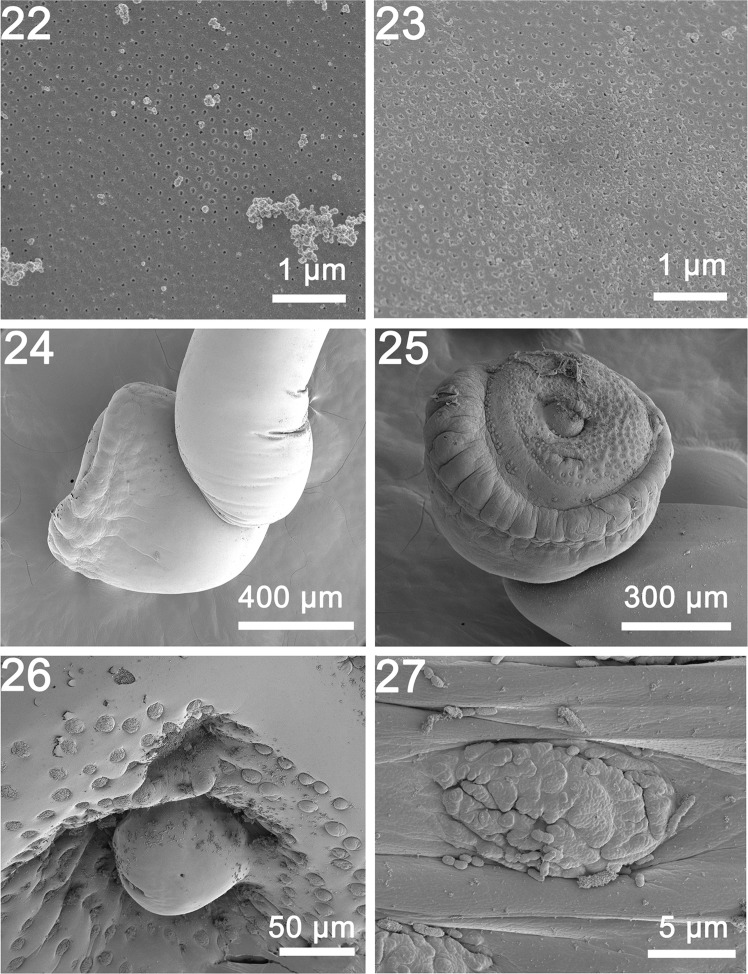
SEM of specimens of *Rhadinorhynchus laterospinosus* from *Auxis rochei* and *Auxis thazard* in the Pacific Ocean off Vietnam. (22, 23) Microspores from the middle and posterior parts of the trunk, respectively. Note the different density and diameter of the pores, also compared with Figure 18 related to differential absorption rates. (24) A lateral view of the bursa. (25) A ventrolateral view of a bursa showing its thick muscular margin and the organization of the outer circle and the central cluster of sensory papillae. (26) A high magnification of the center of the bursa showing the terminal genitalia surrounded by close circles of sensory papillae. This organization is species-specific. (27) A higher magnification of one sensory papilla made up of small units embedded in elliptic depression.

General: With characters of the genus *Rhadinorhynchus* Lühe 1911. Trunk relatively long, uniformly cylindrical, with electron dense micropores that vary in diameter and distribution by region (Figs. 18, 22 and 23), spinose anteriorly in two regions bridged with lateral spines within range of proboscis receptacle (Figs. 1 and 17). Counts of spines on one side of trunk. Trunk spines and all other structures vary by position, host species and worm sex (Table 2). Trunk spines (Fig. 19) larger in females (Fig. 2) than in males. Anterior spines in complete circle with 1–4 dorsal, 1–4 ventral and 1–4 per circle at middle in males and females (Figs. 1 and 17). Posterior ventral spines 0–13 and lateral spines more numerous, 1–31. Length of anterior trunk spines 31–73. Posterior ventral spines larger, 32–95 long. Posterior lateral spines 22–82 long. Posterior trunk spines larger at middle. Proboscis long, cylindrical, straight, gradually widening anteriorly (Figs. 1 and 10) with posterior sac-like membrane evaginating into receptacle (Fig. 29), and 15–19 longitudinal alternating rows of 21–26 hooks each varying with host species (Table 2). Dorsal hooks slightly shorter and more slender that stouter and more sharply curved ventral hooks (Figs. 6 and 7). Hooks slightly arched (Fig. 12) with thin grooved cortical layer and thick core (Figs. 13 and 14), smallest anteriorly, largest at middle, gradually smaller posteriorly except at basal circle of abruptly larger hooks (Figs. 10–12 and 29). Hook roots simple, markedly shorter than blades, directed posteriorly (Figs. 6 and 7). Neck prominent, slightly longer than wide posteriorly, with paired sensory pores (Figs. 15, 16 and 29). Proboscis receptacle double-walled, about twice as long as proboscis with cephalic ganglion near its middle. Lemnisci digitiform, equal, uniformly broad throughout, slightly shorter than receptacle (Fig. 1). Gonopore terminal in males but subterminal in females at level of posterior abrupt narrowing of trunk.

Males (based on 30 adults with sperm from *A. rochei* and *A. thazard*). Trunk 4.75–11.25 (7.04) mm long by 0.35–0.80 (0.56) mm wide at middle. See Table 2 for position, distribution and sizes of trunk spines. Proboscis 1.00–1.67 (1.30) long by 0.17–0.23 (0.20) mm wide anteriorly. See Table 2 for measurements of proboscis hooks. Neck 200–350 (277) long by 175–250 (217) wide posteriorly. Proboscis receptacle 1.62–3.45 (2.34) mm long by 0.14–0.35 (0.23) mm wide. Lemnisci 1.50–2.50 (1.87) mm long by 0.11–0.23 (0.15) mm wide. Reproductive system in posterior half of trunk in contiguous structures with genitalia opening into bursa. Testes ovoid; anterior testis 0.59–1.75 (1.05) mm long by 0.22–0.52 (0.36) mm wide, larger than posterior testis 0.47–1.50 (0.89) mm long by 0.22–0.57 (0.36) wide. Cement glands four, rod-shaped, in two contiguous pairs, each with one tubular giant nucleus (Figs. 1, 3 and 30). Anterior glands 0.31–1.09 (0.73) mm long by 0.14–0.32 (0.20) wide; posterior glands 0.36–1.25 (0.74) mm long by 0.11–0.26 (0.17) mm wide. Individual cement gland ducts surround prominent fusiform Saefftigen’s pouch, 0.42–1.27 (0.89) mm long by 0.15–0.26 (0.21) mm wide, anteriorly (Fig. 3) and joining its genital terminalia (Figs. 26 and 31) at thick-walled bursa (Figs. 24 and 25). Bursa with many elaborate sensory papillae (Fig. 27) at center and in outer rings (Figs. 25 and 33), 208–775 (569) long by 416–831 (625) wide.

**Figures 28–33:**
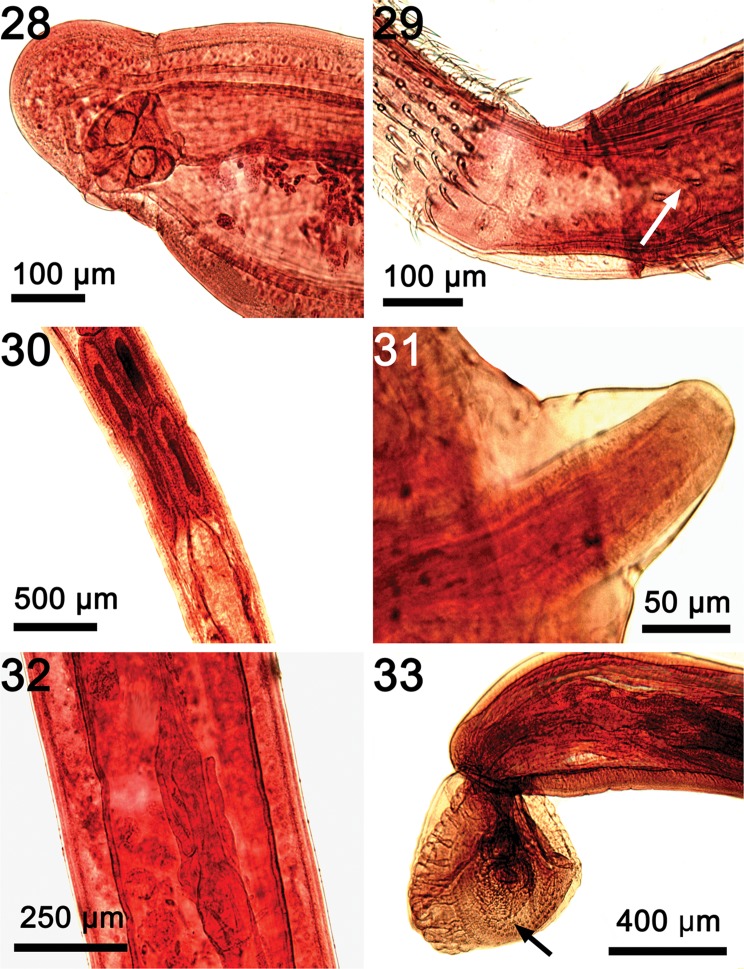
Microscopical images of some internal structures as seen in their natural state not readily demonstrable in line drawings of specimens of *Rhadinorhynchus laterospinosus* from *Auxis rochei* and *Auxis thazard* in the Pacific Ocean off Vietnam. (28) A sub-ventral vagina at the constriction of posterior end of trunk of a female. (29) The posterior loop of a thin sac (arrow) emerging from the insertion of the proboscis receptacle at the base of the proboscis. (30) The four tubular cement glands with their long nuclei just anterior to Saefftigen’s pouch. (31) The penis emerging from the bursa of one specimen. (32) Uterine bell in one female. Note the unequal sides of the bell. (33) The posterior end of one male showing the bursa with rings of sensory papillae (arrow).

Females (based on 32 mature females with eggs and ovarian balls from *A. rochei* and *A. thazard*). Trunk 7.80–21.25 (14.41) mm long by 0.35–1.00 (0.59) mm wide at middle. See Table 2 for position, distribution and sizes of trunk spines. Proboscis 1.25–1.90 (1.64) mm long by 0.17–0.30 (0.24) mm wide anteriorly. See Table 2 for measurements of proboscis hooks. Neck 200–375 (315) long by 109–300 (315) long by 109–300 (233) wide posteriorly. Neck 200–375 (315) long by 109–300 (233) wide posteriorly. Proboscis receptacle 2.24–3.95 (3.14) mm long by 0.17–0.37 (0.25) mm wide. Lemnisci 2.29–3.64 (2.83) mm long by 0.14–0.27 (0.19) wide. Posterior end bluntly pointed with subterminal laterally slit lipless gonopore (Figs. 20). Reproductive system 2.18–5.50 (3.67) mm long; 25% of trunk length (Fig. 9) with well-developed vagina (Figs. 8 and 28), very long and broad uterus, small and elongated uterine bell with unequal walls and two central rod-shaped elongate tubes (Figs. 5 and 32) extending into body cavity, and no uterine bell glands. Eggs fusiform with prominent polar prolongation of fertilization membrane (Figs. 4 and 21), 57–68 (63) long by 12–18 (15) wide.

#### Remarks

The present report represents an expansion of our understanding of *R. laterospinosus* since its description from only one female in 2011 [8] from a trigger fish, *Balistes* sp. from the northern Pacific coast of Vietnam at Cat Ba Island, Halong Bay, Gulf of Tonkin. The single female had a proboscis with 18 longitudinal rows of 24 hooks each, and eight ventral and 18 lateral spines in the posterior field of trunk spines connecting anteriorly with the anterior field of trunk spines. The collection of over 200 specimens from eight additional hosts along the Pacific coast of Vietnam provided an opportunity to describe males, lemnisci, the female reproductive system, and eggs for the first time, and to clarify the dorso-ventral differentiation of proboscis hooks that were inaccurately declared as “similar in shape and size, and in their posteriorly directed angle of projection from proboscis” [8] with the availability of more specimens for study. The new description made it possible to examine the relationship between host species and the expression of certain morphometric parameters. Specimens from *A. thazard* had larger size of trunk, some proboscis hooks, proboscis receptacle, testes, anterior and posterior cement glands, and Saefftigen’s pouch, but relatively fewer and smaller trunk spines than specimens from *A. rochei* (Table 2).

### Energy dispersive X-ray analysis (EDXA)

**Figure 34:**
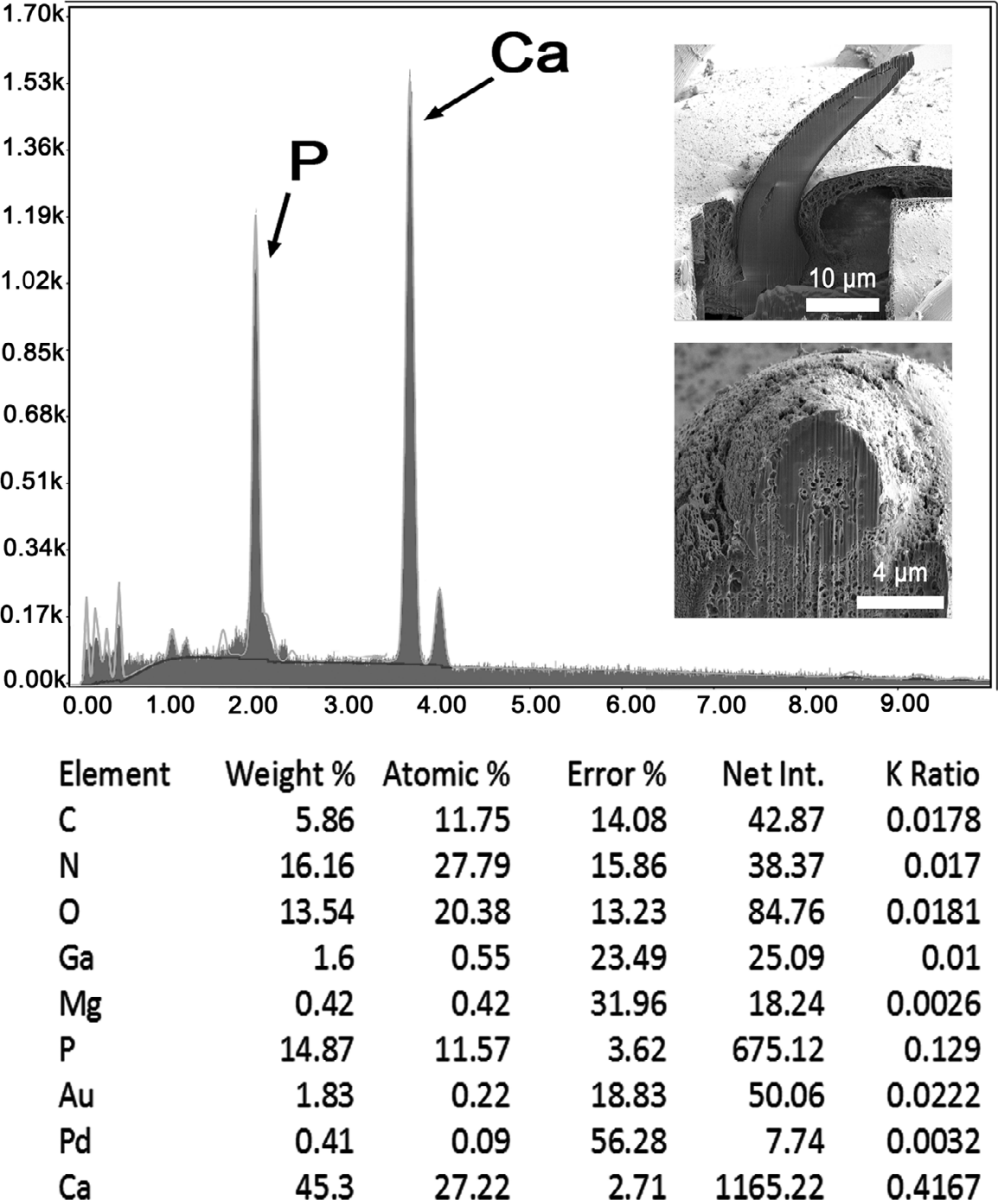
Energy Dispersive X-ray spectrum of the base center of a large hook of a *Rhadinorhynchus laterospinosus* specimen showing high levels of calcium and phosphorus (see Table 3). Insert: SEM of a lateral and cross gallium cut hook.

**Figure 35:**
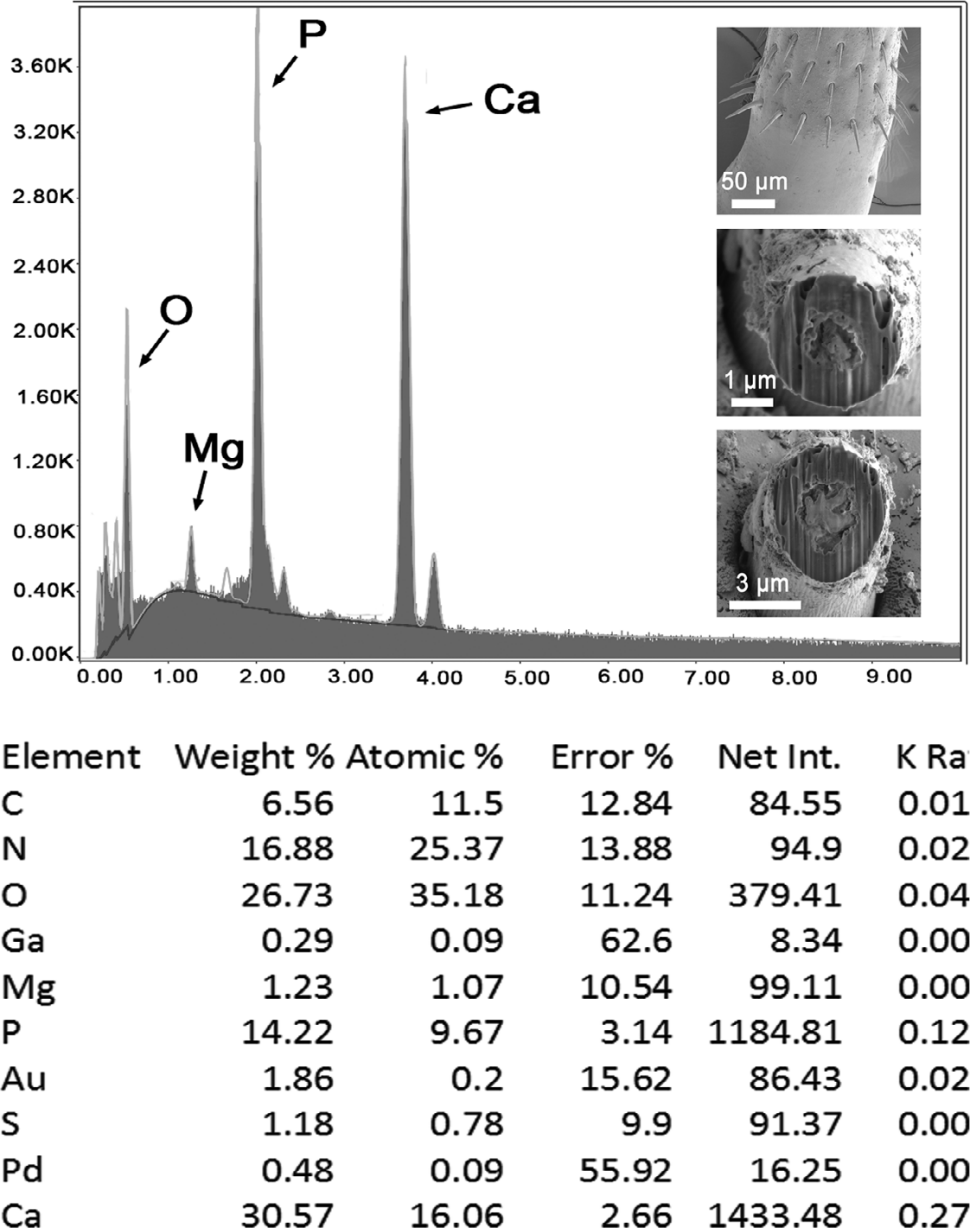
Energy Dispersive X-ray spectrum of the tip of a small hook of a *Rhadinorhynchus laterospinosus* specimen showing high levels of calcium and phosphorus but less calcium than large hooks (see Table 4). Insert: SEM of posterior hooks and hook tips in cross gallium cuts.

**Figure 36:**
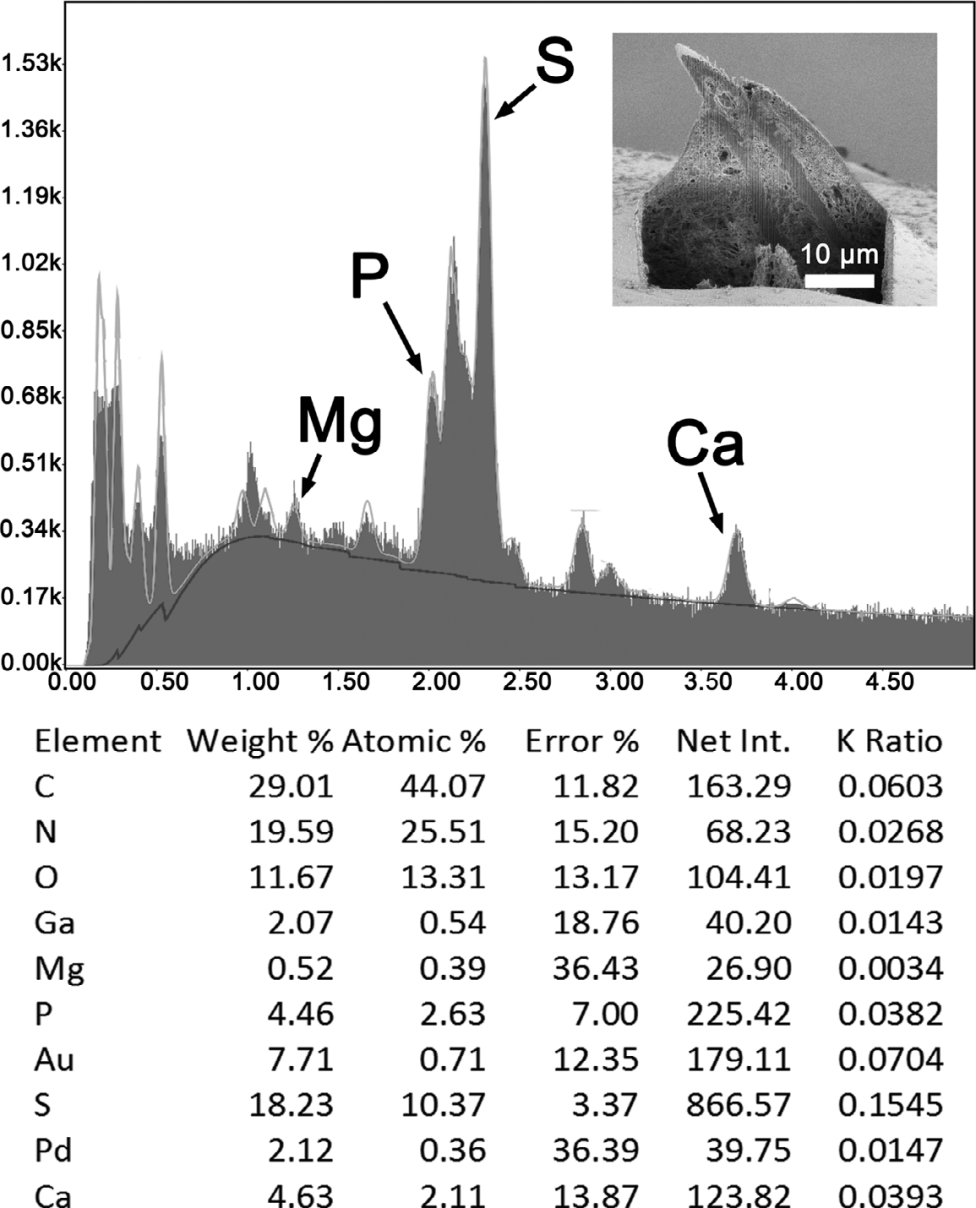
Energy Dispersive X-ray spectrum of the tip of a trunk spine of a *Rhadinorhynchus laterospinosus* specimen showing high levels of sulfur (see Table 5). Insert: SEM of a spine in lateral gallium cut.

We report the X-ray scans and metal composition of large and small proboscis hooks (Figs. 34, 35 and Tables 3, 4) and trunk spines (Table 5 and Fig. 36) of *R. laterospinosus* that were cut with a gallium beam (LMIS) and viewed with a dual beam scanning electron microscope with X-ray capabilities (EDXA). There are variable levels of calcium, phosphorus, and sulfur depending on the type of hook and whether readings are made at the base, core, tip or edge of hooks. Other common elements of living organisms (carbon and oxygen) and elements used for specimen preparation (gallium, palladium, gold) are not included in the analysis. In large hooks, the calcium and phosphorus levels were highest at the center of the hook base (Table 3 and Fig. 34). In small hooks, calcium and phosphorus were highest at hook tips (Table 4 and Fig. 35). Sulfur was high in both spine tip and base compared to calcium and phosphorus (Table 5, Fig. 36).

**Table 3:** X-ray scans for chemical elements of a Gallium cut (LMIS) large hook of *Rhadinorhynchus laterospinosus.*

Elements*	Hook tip edge	Hook tip center	Mid hook edge	Mid hook center	Hook base edge	Hook base center
Magnesium (Mg)	0.00	0.00	0.15	0.59	0.33	0.42
Phosphorus (P)	0.96	0.87	3.27	9.78	7.21	14.87
Sulfur (S)	11.96	15.39	16.59	8.96	12.61	0.00
Calcium (Ca)	2.02	2.04	6.64	22.11	13.90	45.30

* Common protoplasmic elements (C, N, O) as well as processing and coating elements (Pd, Au, Ga) are not included. List in cut%.

**Table 4:** X-ray scans for chemical elements of a Gallium cut small hook at the base of the proboscis of *Rhadinorhynchus laterospinosus.*

Elements*	Hook tip edge	Hook tip center	Hook base edge
Magnesium (Mg)	0.01	1.23	0.02
Phosphorus (P)	1.61	14.22	5.00
Sulfur (S)	17.88	1.18	17.65
Calcium (Ca)	2.35	30.57	9.82

* Common protoplasmic elements (C, N, O) as well as processing and coating elements (Pd, Au, Ga) are not included. List in cut%.

**Table 5:** X-ray scans for chemical elements of a Gallium cut spine of *Rhadinorhynchus laterospinosus.*

Elements*	Spine tip	Spine base
Magnesium (Mg)	2.07	0.49
Phosphorus (P)	4.46	3.67
Sulfur (S)	18.23	11.64
Calcium (Ca)	4.63	3.48

* Common protoplasmic elements (C, N, O) as well as processing and coating elements (Pd, Au, Ga) are not included. List in cut%.

### Molecular results

Three partial 18S rDNA (741–767 nt) and four *cox*1 (606–622 nt) sequences were generated from four adult specimens (two males and two females) of *R. laterospinosus*. While 18S rDNA sequences were identical (only the longest one was thus included in the corresponding phylogenetic trees), intraspecific sequence divergence for *cox*1 ranged between 0.008 and 0.018% (5–11 nt difference).

**Figure 37:**
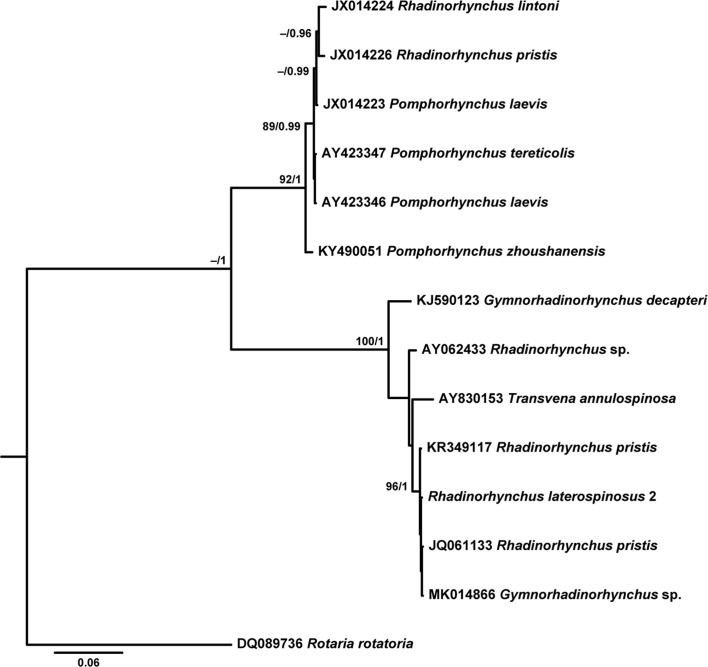
Bayesian inference (BI) phylogram reconstructed using a newly generated 18S rDNA sequence for *Rhadinorhynchus laterospinosus* and retrieved sequences from GenBank for *Rhadinorhynchus* and the closest-related sequences to members of this genus. Outgroup: *Rotaria rotatoria*. Nodal support from maximum likelihood (ML) and Bayesian inference (BI) analyses are indicated as ML/BI. Bootstrap values lower than 70 and posterior probability values lower than 0.95 are omitted. The scale-bar indicates the expected number of substitutions per site.

**Figure 38:**
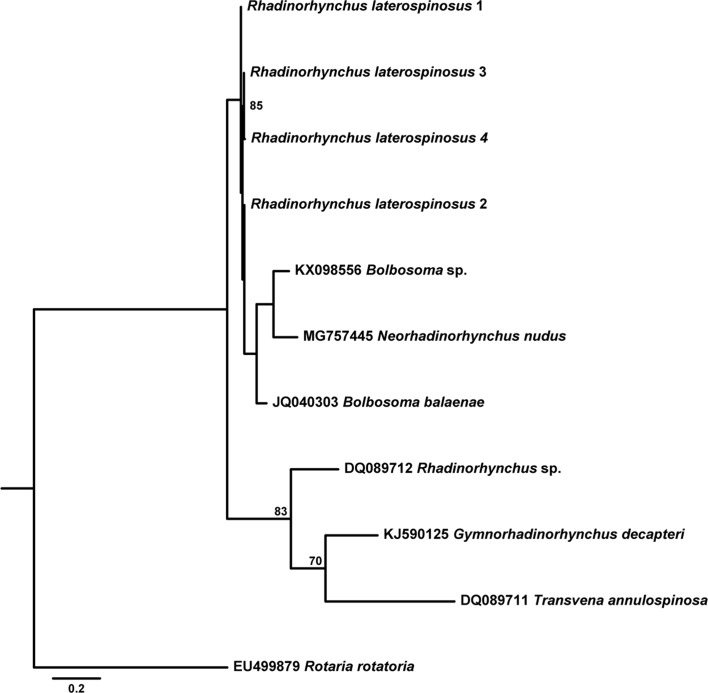
Maximum likelihood (ML) phylogram reconstructed using four newly generated *cox*1 sequences for *Rhadinorhynchus laterospinosus* and retrieved sequences from GenBank for *Rhadinorhynchus* and the closest-related sequences to members of this genus. Outgroup: *Rotaria rotatoria*. Bootstrap values lower than 70 are omitted. The scale-bar indicates the expected number of substitutions per site.

**Figure 39:**
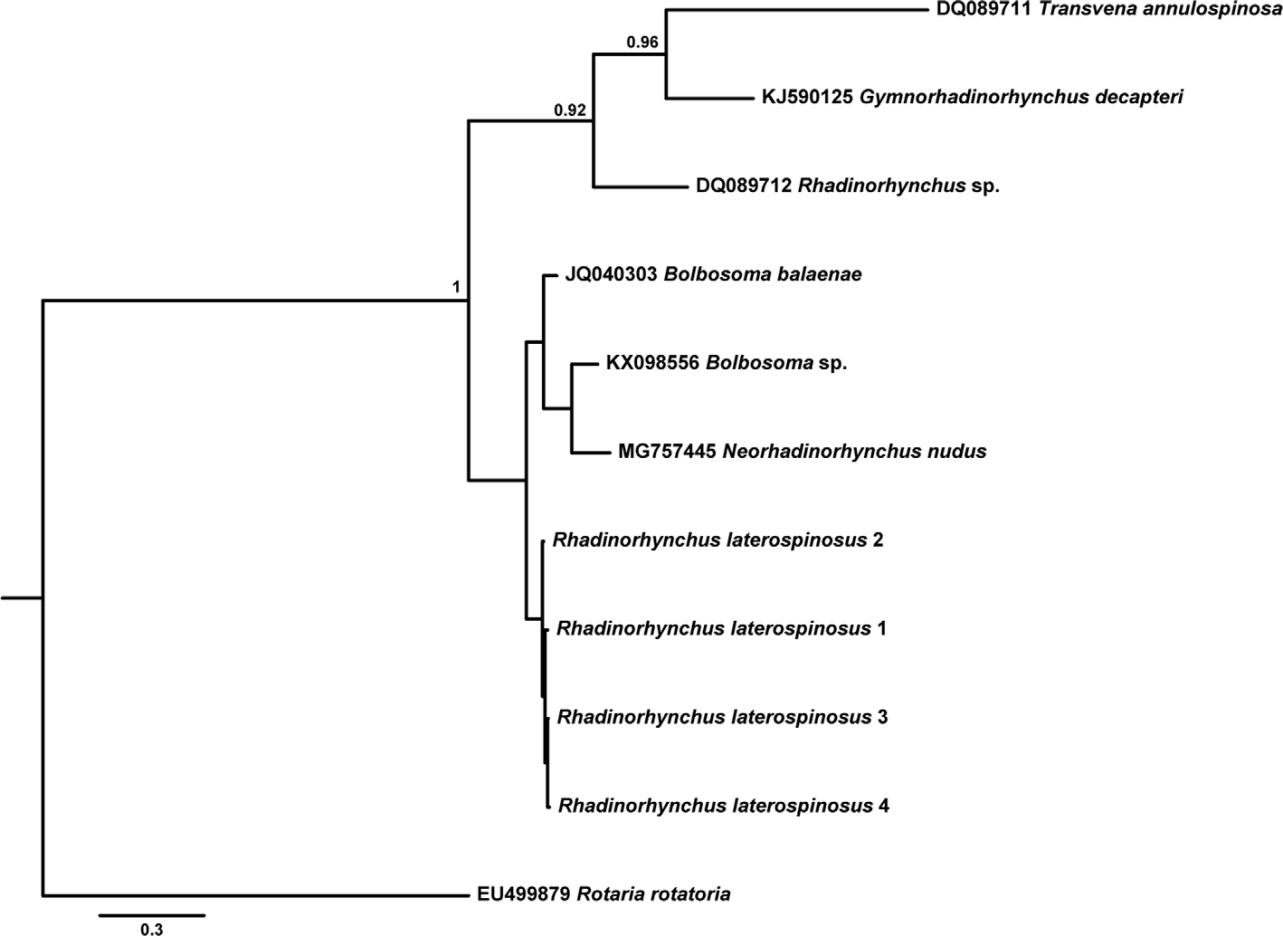
Bayesian inference (BI) phylogram reconstructed using four newly generated *cox*1 sequences for *Rhadinorhynchus laterospinosus* and retrieved sequences from GenBank for *Rhadinorhynchus* and the closest-related sequences to members of this genus. Outgroup: *Rotaria rotatoria*. Posterior probability values lower than 0.95 are omitted. The scale-bar indicates the expected number of substitutions per site.

Table 6 provides data for the sequences retrieved from GenBank and used in the phylogenetic analyses based on the two alignments. While both ML and BI algorithms produced trees with identical topology for the 18S gene (Fig. 37), a slightly different topology was observed for the *cox*1 gene (Figs. 38 and 39).

**Table 6:** Data for the sequences belonging to Paleacanthocephala retrieved from GenBank and included in the phylogenetic analyses.

Order (Family)	Species	GenBank ID		Location	Reference
		18S	*cox*1		
Echinorhynchida (Rhadinorhynchidae)	*Rhadinorhynchus pristis* (Rudolphi, 1802)	KR349117		Minho and Mondego rivers (Western Iberian Peninsula)	Bao et al. [20]
		JQ061133		Atlantic Ocean (Vigo, Spain)	Gregori et al. [29]
		JX014226		Indian Ocean (Java, Indonesia)	Verweyen et al. [48]
	*Rhadinorhynchus lintoni* Cable and Linderoth, 1963	JX014224		Pacific Ocean (Hawaii, USA)	Verweyen et al. [48]
	*Rhadinorhynchus* sp.	AY062433		Unknown	García-Varela et al. [24]
	*Rhadinorhynchus* sp.		DQ089712	Unknown	García-Varela and Nadler [26]
Echinorhynchida (Cavisomidae)	*Neorhadinorhynchus nudus* (Harada, 1938) Yamaguti, 1939		MG757445	Pacific Ocean – South China Sea (Shanwei, China)	Li et al. [38]
Echinorhynchida (Gymnorhadinorhynchidae)	*Gymnorhadinorhynchus decapteri* Braicovich, Lanfranchi, Farber, Marvaldi, Luque and Timi, 2014	KJ590123	KJ590125	Atlantic Ocean (Cabo Frío, Brazil)	Braicovich et al. [21]
	*Gymnorhadinorhynchus* sp.	MK014866		Pacific Ocean (Japan)	Steinauer et al. [45]
Echinorhynchida (Transvenidae)	*Transvena annulospinosa* Pichelin and Cribb, 2001	AY830153		Unknown	García-Varela and Nadler [25]
			DQ089711	Unknown	García-Varela and Nadler [26]
Echinorhynchida (Pomphorhynchidae)	*Pomphorhynchus laevis* (Zoega in Müller, 1776)	JX014223		Atlantic Ocean – Baltic Sea	Verweyen et al. [48]
		AY423346		Ouche river (Dijon, France)	Perrot-Minot [41]
	*Pomphorhynchus tereticollis* (Rudolphi, 1809)	AY423347		Ouche river (Dijon, France)	Perrot-Minot [41]
	*Pomphorhynchus zhoushanensis* Li, Chen, Amin and Yang, 2017	KY490051		Pacific Ocean – South China Sea (Zhoushan Islands, China)	Li et al. [37]
Polymorphida (Polymorphidae)	*Bolbosoma balaenae* (Gmelin, 1790)		JQ040303	Atlantic Ocean (Vigo, Spain)	Gregori et al. [28]
	*Bolbosoma* sp.		KX098556	Atlantic Ocean – Gulf of Mexico	Andres et al. [18]

Phylogenetic analyses based on 18S rDNA gene demonstrated the strong association between *R. laterospinosus* and other representatives of the genus (*R. pristis*) with maximum support (0.000–0.013%, 0–10 nt difference), but also with a sequence classified as *Gymnorhadinorhynchus* sp., which showed no differences with the present sequence (0%, 0 nt). Sequences belonging to *Gymnorhadinorhynchus decapteri* Braicovich, Lanfranchi, Farber, Marvaldi, Luque and Timi, 2014 and *Transvena annulospinosa* Pichelin and Cribb, 2001 were also included in this clade (0.024–0.039%, 18–29 nt difference from newly generated sequences). Two sequences belonging to *R. pristis* (Rudolphi, 1802) and *R. lintoni* Cable and Linderoth, 1963 (0.184–0.185%, 138–139 nt difference from newly generated ones) remained on a separate clade and were strongly associated with members of *Pomphorhynchus*.

According to phylogenetic analyses based on the *cox*1 gene, the four newly generated sequences for the *R. laterospinosus* grouped, with low support, with a clade formed by representatives of *Bolbosoma* and the species *Neorhadinorhynchus nudus* (Harada, 1938) Yamaguti, 1939 (0.076–0.141%, 39–72 nt difference), and remained apart from a third clade which included the only available published sequence on this gene for *Rhadinorhynchus* (0.238–0.242%, 122–124 nt difference from newly generated sequences).

## Discussion

### Morphometric comparisons

The observed relationship between host species and size and even shape of acanthocephalans observed in this study (Table 2) has been previously demonstrated for other acanthocephalans including *Echinorhynchus salmonis* Müller, 1784 whose variability in the size of taxonomically important structures such as the trunk, proboscis hooks, proboscis, testes, etc. has been attributed to host species. Such relationships have been reported in Lake Michigan where male and female specimens from bloater, *Coregonus hoyi* (Gill) (Salmonidae) achieved not only larger size but also different body form (broad anteriorly) compared to the slender specimens from rainbow smelt, *Osmerus mordax* (Mitchell) (Osmeridae) [17]. The larger and heavier worms from bloater invariably showed a higher regression coefficient (adjusted coefficient of determination) compared to those from smelt in all characters including size of trunk, proboscis, longest proboscis hooks, receptacle, testes, lemnisci, and eggs. The taxonomic implications of this variability were discussed (Amin and Redlin, 1980). Earlier, Amin [1] demonstrated a similar relationship for *Acanthocephalus dirus* (Van Cleave, 1931) Van Cleave and Townsend, 1936 in Wisconsin fishes. Females of the same developmental stage recovered during the same period were found to have attained larger sizes in certain hosts than in others with the largest females being found in *Lepomis macrochirus* Rafinesque. The size of the trunk in males was also found to follow the same pattern. Similarly, testes also attained a larger size in males recovered from *Catostomus commersonii* Lacépède (Catostomidae) than in males from *Semotilus atromaculatus* (Mitchill) (Cyprinidae). Amin [1] stated that these size variations “result from differential growth rates of these worms in the various host intestinal environments (and) are probably mediated by certain host specific factors.”

### Distribution

Amin [2] and Amin et al. [8] recognized 38 valid species of *Rhadinorhynchus* and invalidated 30 others. Only five more species of *Rhadinorhynchus* were described since, four from marine fishes off Australia [43] and *Rhadinorhynchus oligospinosus* Amin and Heckmann, 2017 off the Peruvian Pacific coast. The 43 valid species of *Rhadinorhynchus* include 20 species from the Pacific Ocean, especially off Australia, Japan, and Vietnam. These species are:

1. *Rhadinorhynchus bicircumspinus* Hooper, 1983 from New South Wales, Australia.
2. *Rhadinorhynchus biformis* Smales, 2014 from Heron Island, Australia.
3. *Rhadinorhynchus carangis* Yamaguti, 1939 from the Seto Inland Sea, Japan.
4. *Rhadinorhynchus chongmingnensis* Huang, Zheng, Deng, Fan et Ni, 1988 from Chongming, China.
5. *Rhadinorhynchus cololabis* Laurs et McCauley, 1964 from Oregon, USA.
6. *Rhadinorhynchus decapteri* Parukhin et Kovalenko, 1976 from Hawaii.
7. *Rhadinorhynchus ditrematis* Yamaguti, 1939 from the Seto Inland Sea, Japan.
8. *Rhadinorhynchus dorsoventrospinosus* Amin, Heckmann, Ha 2011 from Halong Bay, Vietnam.
9. *Rhadinorhynchus johnstoni* Golvan, 1969 from South Australia.
10. *Rhadinorhynchus laterospinosus* Amin, Heckmann, Ha, 2011 from Halong Bay, Vietnam.
11. *Rhadinorhynchus oligospinosus* n. sp. from Port of Chicama, La Libertad, Peru.
12. *Rhadinorhynchus ornatus* Van Cleave, 1918 from the Atlantic coast of the USA, Japan, and the Pacific Ocean off South America.
13. *Rhadinorhynchus pichelinae* Smales, 2014 from Point Peron, Western Australia.
14. *Rhadinorhynchus polydactyli* Smales, 2014 from Moreton Bay, Queensland, Australia.
15. *Rhadinorhynchus polynemi* Gupta and Lata, 1967 from India and north-east Australia.
16. *Rhadinorhynchus pomatomi* Smales, 2014 from New Brighton, New South Wales, Australia.
17. *Rhadinorhynchus selkirki* Van Cleave, 1920 from Juan Fernandez Island, Chili.
18. *Rhadinorhynchus seriolae* (Yamaguti, 1963) Golvan, 1969 from Japan and Australia.
19. *Rhadinorhynchus trachuri* Harada, 1935 from a Tokyo market, Japan.
20. *Rhadinorhynchus zhukovi* Golvan, 1969 from the Kuril Islands, Japan–Russia.

### Morphological comparisons

Morphologically, *Rhadinorhynchus stunkardi* Gupta et Fatma, 1987 from India is the only other species of *Rhadinorhynchus* that has lateral trunk spines connecting the anterior and posterior fields of trunk spines like *R. laterospinosus*. *Rhadinorhynchus stunkardi*, however, has only 3–4 posterior trunk spines on the ventral side, only 8–10 proboscis hook rows each with 24–26 small hooks that reach a maximum length of only 46, considerably larger eggs, 120–150 × 25–28, and a terminal gonopore [31].

### Energy dispersive X-ray analysis (EDXA)

The results of the X-ray analysis (Tables 3–5 and Figs. 34–36) of gallium cut hooks and spines of *R. laterospinosus* show that the large hooks in the mid-proboscis and the small posterior hooks had a high level of sulfur at the tip edge, which is consistent with the base of the hooks. This element along with calcium and phosphorus aid in the mineralization and hardening of the outer layer of hooks, similar to the enamel layer of the mammalian tooth (calcium phosphate apatite) [32]. The base center of large hooks shows increased levels of calcium (45.30%) and phosphate ions (14.87) (Table 3), comparable to the inner core of mammalian teeth [6]. Sulfur levelsshowed a higher differential concentration at the edge than the middle of cut hooks (Tables 3 and 4). This element is part of the prominent outer layer of most acanthocephalan hooks and is a major contributor to the hardening process of this attachment structure. There is a difference in the distribution of calcium ions in the smaller hooks in relation to large hooks, this level being highest in the core and base of large hooks (45/30%) but highest at the tip of small hooks (30.57%) (Tables 3 and 4). A similar EDAX study of the proboscis hooks of *Echinorhynchus baeri* Kostylew, 1928 showed that large hooks have higher calcium, phosphorus, and sulfur than miniature rootless hooks [6]. Comparable patterns for the numerous trunk spine gallium cuts (Table 5) demonstrate the rigid nature of the spine which is explained by the X-ray scans (Fig. 36). There is a reasonably high level of phosphorus, calcium and especially sulfur at the tip (18.23%) and base (11.64%) of the spine, which have mineralized to form the rigid support. The X-ray scans of the gallium cut hooks and spines help explain the morphological nature of *R. laterospinosus* and identify its unique “personality” [44]. The uniqueness of the metal analysis as expressed by X-ray scans appears to be species-specific and can be regarded as a fingerprint of key diagnostic value that is just as important as molecular analysis. This was well demonstrated in the study of *Rhadinorhynchus oligospinosus* Amin and Heckmann, 2017 from mackerels in the Pacific Ocean off Peru [5], among others.

### Micropores

The presence of micropores on various trunk regions of specimens of *R. laterospinosus* (Figs. 18, 22 and 23) suggests differential nutrient absorption related to the diameter and distribution of micropores as appears to be the case in practically all acanthocephalans. We have documented this phenomenon in 16 species of acanthocephalans [33] and a few more since. The functional aspects of micropores in a few other acanthocephalan species including *Rhadinorhynchus ornatus* Van Cleave, 1918, *Polymorphus minutus* (Goeze, 1782) Lühe, 1911, *Moniliformis moniliformis* (Bremser, 1811) Travassos (1915), *Macracanthorhynchus hirudinaceus* (Pallas, 1781) Travassos (1916, 1917), and *Sclerocollum rubrimaris* Schmidt and Paperna, 1978 were reviewed earlier [7]. The micropore canals appear to be continuous with canalicular crypts that constitute a huge increase in external surface area implicated in nutrient uptake [7].

### Molecular analysis

To date, genetic data have been provided for only three species of *Rhadinorhynchus*: *R. laterospinosus* (present results), *R. pristis*, and *R. lintoni* (see Table 6 for references). The scarcity of molecular profiles described for this genus poses difficulties for correctly determining relationships among its members and with other genera, and adds importance to the new molecular data presented herein.

The lack of congruence between taxonomy and evolutionary history within *Rhadinorhynchus* observed in the 18S rDNA- and *cox*1-derived phylogenies has been noted previously by other authors based on morphological [40] and genetic markers (18S and 28S rDNA and *cox*1 genes) [21, 29]. Indeed, while the sequences provided for *R. pristis* and *R. lintoni* [48] form a strongly supported clade with members of the genus *Pomphorhynchus* in the 18S rDNA-derived phylogram, the rest of the available *Rhadinorhynchus* sequences (including newly generated ones) form a clearly separate group that also includes sequences from *T. annulospinosa* and *G. decapteri*. This pattern was highlighted previously [21, 29]. While Gregori et al. [29] questioned the genetic identification of the specimens characterized by Verweyen et al. [48], Braicovich et al. [21] attributed this pattern to incorrect assignment to *Rhadinorhynchus* by García-Varela et al. [24]. In fact, in their revision of the genus, Amin et al. [8] classified *R. pristis* and *R. lintoni* from Atlantic and Mediterranean waters as invalid species, which supports the view by Gregori et al. [29] given that specimens collected by Verweyen et al. [48] were from Pacific waters. This solves the paraphyly “problem” observed in these previous phylogenies and in the ones presented herein based on the 18S rDNA gene. Because previously described 18S rDNA *Rhadinorhynchus* sequences [20, 29] and present results group with those provided by García-Varela et al. [24], the suggestion by Braicovich et al. [21] of a misidentification by the latter author could be ruled out. Another specimen belonging to the same clade has recently been classified into the genus *Gymnorhadinorhynchus* [45]. The null difference between this sequence and the newly generated one for *R. laterospinosus* points to a need for reclassification of this *Gymnorhadinorhynchus* sp. specimen most probably into the genus *Rhadinorhynchus*.

The outcome of the phylogenetic analysis based on the *cox*1 gene is less complete than the 18S rDNA-based one due to the near absence of *cox*1 gene sequences for *Rhadinorhynchus* in GenBank. Even so, it shows conflictive relationships for members of this genus, with present sequences forming a sub-clade within a group including *Bolbosoma* members and *N. nudus*, apart from the group formed by *Rhadinorhynchus* sp., *T. annulospinosa*, and *G. decapteri*. Although the goal of the present study is not to discuss the higher level classification of Paleacanthocephala, the inclusion of the echinorhynchid *N. nudus* within the Polymorphidae (i.e. *Bolbosoma*) further demonstrates the extent of these inconsistencies at the supra-familiar level. In fact, the paraphyly within the palaeacanthocephalan at the family level is well established [25, 34, 40, 48], which highlights the existing problems with their taxonomic arrangement and points to the need for a reclassification based on better morphological, ecological and molecular characterization of their members.

To summarize, following the 18S rDNA-based analysis, a single clade including all the valid species of the genus *Rhadinorhynchus* described up until now is recognized. However, *Rhadinorhynchus* relationships in phylogenetic analysis based on *cox*1 sequences are not so clear, mostly due to the lack of published sequences of this gene so far. Conflicting relationships with other genera (i.e. *Gymnorhadinorhynchus*, *Transvena*, *Bolbosoma* and *Neorhadinorhynchus*) are apparent in both phylogenies, underlining the importance of elucidating relationships within the Paleacanthocephala in future studies.
